# Altered spread of waves of activities at large scale is influenced by cortical thickness organization in temporal lobe epilepsy: a magnetic resonance imaging–high-density electroencephalography study

**DOI:** 10.1093/braincomms/fcad348

**Published:** 2023-12-14

**Authors:** Gian Marco Duma, Giovanni Pellegrino, Giovanni Rabuffo, Alberto Danieli, Lisa Antoniazzi, Valerio Vitale, Raffaella Scotto Opipari, Paolo Bonanni, Pierpaolo Sorrentino

**Affiliations:** Epilepsy Unit, IRCCS E. Medea Scientific Institute, Conegliano 31015, Italy; Epilepsy Program, Schulich School of Medicine and Dentistry, Western University, London N6A5C1, Canada; Institut de Neurosciences des Systèmes, Aix-Marseille Université, Marseille 13005, France; Epilepsy Unit, IRCCS E. Medea Scientific Institute, Conegliano 31015, Italy; Epilepsy Unit, IRCCS E. Medea Scientific Institute, Conegliano 31015, Italy; Department of Neuroscience, Neuroradiology Unit, San Bortolo Hospital, Vicenza 36100, Italy; Department of Neuroscience, Neuroradiology Unit, San Bortolo Hospital, Vicenza 36100, Italy; Epilepsy Unit, IRCCS E. Medea Scientific Institute, Conegliano 31015, Italy; Institut de Neurosciences des Systèmes, Aix-Marseille Université, Marseille 13005, France; Department of Biomedical Sciences, University of Sassari, Sassari 07100, Italy

**Keywords:** temporal lobe epilepsy, neuronal avalanches, structural covariance network, connectome

## Abstract

Temporal lobe epilepsy is a brain network disorder characterized by alterations at both the structural and the functional levels. It remains unclear how structure and function are related and whether this has any clinical relevance. In the present work, we adopted a novel methodological approach investigating how network structural features influence the large-scale dynamics. The functional network was defined by the spatio-temporal spreading of aperiodic bursts of activations (neuronal avalanches), as observed utilizing high-density electroencephalography in patients with temporal lobe epilepsy. The structural network was modelled as the region-based thickness covariance. Loosely speaking, we quantified the similarity of the cortical thickness of any two brain regions, both across groups and at the individual level, the latter utilizing a novel approach to define the subject-wise structural covariance network. In order to compare the structural and functional networks (at the nodal level), we studied the correlation between the probability that a wave of activity would propagate from a source to a target region and the similarity of the source region thickness as compared with other target brain regions. Building on the recent evidence that large-waves of activities pathologically spread through the epileptogenic network in temporal lobe epilepsy, also during resting state, we hypothesize that the structural cortical organization might influence such altered spatio-temporal dynamics. We observed a stable cluster of structure–function correlation in the bilateral limbic areas across subjects, highlighting group-specific features for left, right and bilateral temporal epilepsy. The involvement of contralateral areas was observed in unilateral temporal lobe epilepsy. We showed that in temporal lobe epilepsy, alterations of structural and functional networks pair in the regions where seizures propagate and are linked to disease severity. In this study, we leveraged on a well-defined model of neurological disease and pushed forward personalization approaches potentially useful in clinical practice. Finally, the methods developed here could be exploited to investigate the relationship between structure–function networks at subject level in other neurological conditions.

## Teaser

Duma *et al*. used innovative methods like subject-wise structural covariance network and neuronal avalanches to study structural and functional brain organization in temporal lobe epilepsy. A coupling was observed between the propagation of aperiodic burst of activity and cortical thickness organization focused in brain regions related to seizure initiation and propagation.

## Graphical Abstract

**Graphical Abstract:**
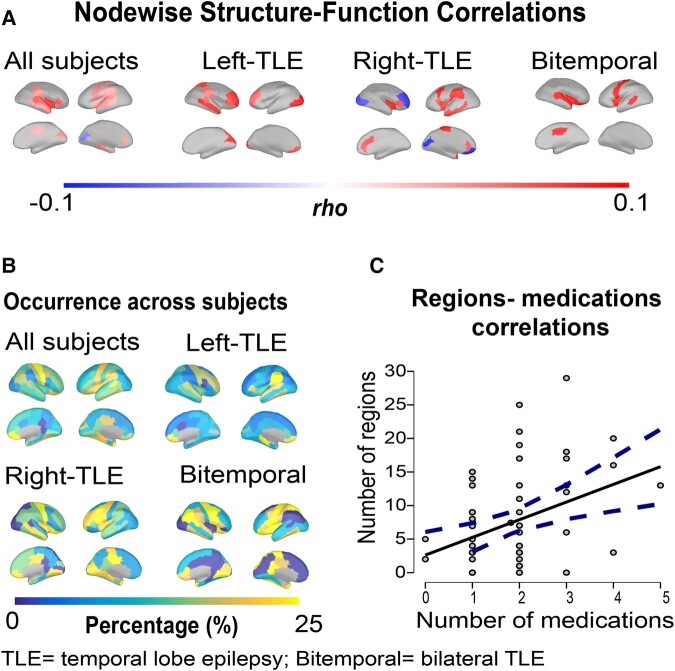

## Introduction

Epilepsy is currently regarded as a network disorder, affecting not only the local node (i.e. the epileptogenic zone; EZ) but also the whole-brain network organization.^1,2^ Alterations have been identified both at the structural and functional levels. Findings from MRI studies have demonstrated that structural alterations are not limited to the EZ patients with epilepsy but, rather, diffused alteration of the cytoarchitectonic and morphological cortical organization is present.^3-6^ In line with these results, neuroimaging and electrophysiological studies have shown patterns of altered functional connectivity impacting the whole brain, both during ictal and interictal activities.^7-10^ However, the relationship between structural and functional alterations is a complex one, which largely remains to be elucidated. Recent studies suggest that including regional heterogeneity, either with respect to morphological, cytoarchitectonics or neuromodulatory information, is fundamental to understand and to model the role of the structural organization in constraining the spatio-temporal dynamics.^11,12^ In relation to this, in the present study, we hypothesized that the distribution of cortical thickness in the brain may be linked to the altered spreading of the large-scale perturbations in relation to epilepsy. To test this hypothesis, we used a multimodal data set made of source-reconstructed high-density electroencephalography (hdEEG) and structural MRI from 59 patients with temporal lobe epilepsy (TLE). The model of TLE is of particular interest because it represents the most diffused type of focal epilepsy.^13^ Leveraging on our previous work on altered avalanche spreading in TLE,^14^ we expect to identify a relationship between the organization of the cortical morphology and the propagation of the activity bursts and specifically so in the epileptogenic network. Moreover, in the light of the relationship between thickness and disease duration,^4^ as well as the exposure to antiseizure medications (ASMs),^15,16^ we explored the dependence of structure–function link in relation to the age of onset, the disease duration and the number of ASMs. To this end, we focused on the avalanche transition matrices (ATMs). The ATMs are a tool to measure how large-scale perturbations, also referred as ‘neuronal avalanches’, spread across the brain. In particular, neuronal avalanches belong to the framework of criticality, which lends itself nicely to the investigation of the microscopic dynamical behaviour of neuronal assemblies and its relation to large-scale alterations such as, in the case of epilepsy, seizure initiation, propagation and termination.^17-19^In particular, the ATMs are a recently developed analytical tool that captures the probability of any two regions being successively recruited by spontaneous neuronal avalanches resulting in a functional network.^11,20,21^ In order to better characterize the link between functional and morphological brain configurations, we adopted a network-level method also for the thickness distribution. To this end, we analysed the structural covariance network (SCN), a measure adopted in the workflow of the ENIGMA.^6^ The SCN describes the existence of correlated anatomical measurements, such as cortical thickness, between pairs of brain regions, proving a network measure of the thickness organization.^22^ By providing information on the morphology heterogeneity of the cortex, the SCN can be used to elucidate the relationship between the structural and functional connectomes. However, as the SCN has been designed as a group metric, it is not trivial to extract subject-wise fashion information. In this work, by generalizing the co-fluctuation framework,^23,24^ we have been able to obtain a subject-specific measure for the SCN (see Materials and methods section) in order to better quantify the individual contributions to the structure–function relationship. This metric will be referred to as subject-wise structural covariance network (sSCN) from here on. We studied the structural–functional relationship by comparing structural and functional matrices, first at the group level and then in a subject-specific fashion. In fact, utilizing the subject-wise investigation, we aimed at capturing fluctuations that may be related with pathology-related variables, which is relevant for the clinical translation of our approach. This is in line with the application of individual network-level measurements together with clinical variables in the framework of personalized diagnosis.^25^

## Materials and methods

### Participants

We retrospectively enrolled 70 patients with temporal lobe epilepsy, who underwent hdEEG for clinical evaluation in 2018–2021 at the Epilepsy and Clinical Neurophysiology Unit, IRCCS Eugenio Medea cited in Conegliano (Italy). The diagnosis workflow included clinical history and examination, neuropsychological assessment, long-term surface video EEG (32 channels) monitoring, hdEEG resting-state recording, brain MRI and PET as an adjunctive investigation in selected cases. The diagnosis of temporal lobe epilepsy was established according to the International League Against Epilepsy (ILAE) guidelines. Eleven subjects were excluded due to the poor quality of the MR images resulting in a sample of 59 patients with TLE [29 left TLE, 17 right TLE, 13 bitemporal TLE (34M, 25F)]. Following the ILAE consensus guidelines,^26^ 7 patients were defined as drug responsive. A description of the patients’ demographic and clinical characteristics is provided in Table 1. The patients underwent neuropsychological assessment, which is described in detail, along with the scores, in the Supplementary materials (see Supplementary Table 2). The study protocol was conducted according to the Declaration of Helsinki and approved by the local ethical committee.

**Table 1:** Demographic and clinical characteristics of the patients with temporal lobe epilepsy

Patients with TLE	Mean ± SD
Age	39.61 ± 17.66
Age of onset	23.36 ± 19.28
Duration of epilepsy (years)	18.78 ± 19.17
Number of antiseizure medications	1.83 ± 1.00
Antiseizure medications	Number
ACT	1
AZM	1
BLB	1
BRV	6
CBZ	15
CLB	5
CZP	2
ESL	12
LCM	15
LEV	8
LTG	5
OXC	4
PB	2
PER	9
VPA	10
ZNS	1
NO-ASMs	2
MRI	
Mesial	Number
HS	11
DNET	1
UKN	8
Amygdala enlargement	6
Anterior (temporal pole)	
FCD	13
Encephalocele	2
Gliosis	2
Anterior + mesial	
FCD + HS	5
Developmental venous anomaly	1
Negative MRI	10

MRI abnormalities are reported by sublobar localization. The continuous variables are reported as mean ± SD. Antiseizure medication abbreviations: ACT, acetazolamide; AZM, acetazolamide; BRV, brivaracetam; CBZ, carbamazepine; CLB, clobazam; CZP, clonazepam; ESL, eslicarbazepine; LCM, lacosamide; LEV, levetiracetam; LTG, lamotrigine; OXC, oxcarbazepine; PB, phenobarbital; PER, perampanel; VPA, valproic acid; ZNS, zonisamide; NO-ASMs, no pharmacological treatment. Abbreviation of the identified anomalies on the MRI: FCD, focal cortical dysplasia; HS, hippocampal sclerosis; DNET, dysembryoplastic neuroepithelial tumours; UKN, unknown.

### Morphological measures and covariance networks

We used the individual MRI anatomy in order to generate individualized head models for the patients with TLE. The anatomical MRI for source imaging consisted of a T_1_-weighted 1 mm isotropic 3D acquisition. All the MRI acquisition details are reported in the supplementary materials (see Supplementary Table 1). The MRI was segmented in the skin, skull and grey matter using the segmentation pipeline of the Computational Anatomy Toolbox (CAT12). Successively, we computed the cortical thickness using CAT12, which yields a morphological value for each vertex of the brain mesh. Cortical data were harmonized across scanners using the Matlab implementation of ComBat.^27^ We used the Desikan–Killiany parcellation,^28^ and then by averaging across all the vertices within each region of the atlas, we obtained the morphological index value $X_{i}(s)\;\;$ for each region *i* of the atlas and for each subject *s*. The SCN of a group of subjects is a matrix which is computed by estimating the inter-regional Pearson’s correlation across subjects of the cortical index between all possible pairs of regions. Each element of the SCN matrix is defined as:


(1) $$\;{\mathrm{SCN}}_{ij}=\langle Z_{i}(s)\cdot Z_{j}(s)\rangle_{s}=\left\langle \frac{X_{i}(s)-\bar{X_{i}(s)}}{\sigma (X_{i}(s))}\cdot \frac{X_{j}(s)-\bar{X_{i}(s)}}{\sigma (X_{j}(s))}\right\rangle s,$$

where $Z_{i}(s)$ denotes the *z*-score of the cortical index $X_{i}(s)$ of region *i*, with $\bar{X_{i}(s)}\;$ and $\sigma (X_{i}(s))$ representing its mean and variance across all subjects *s*, respectively. The formula above defines the Pearson’s correlation coefficient for a pair *ij* of cortical indices, i.e. the product of *z*-scored cortical indices, averaged across all subjects. Thus, the construction of the SCN relies on the identification of spatial patterns of morphometric similarities between brain regions within a group of subjects. The SCN matrix can be expressed at the single-subject level by defining the sSCN as:


(2) $$\;\mathrm{sSC}N_{ij}(s)=Z_{i}(s)\cdot Z_{j}(s)=\;\frac{X_{i}(s)-\bar{X_{i}(s)}}{\sigma (X_{i}(s))}\cdot \frac{X_{j}(s)-\;X_{j}(s)}{\sigma (X_{j}(s))}.$$

This way, the SCN is the result of averaging across individual sSCN(s), i.e.:


(3) $${\text{SCN}}_{ij}=\langle {\text{sSCN}}_{ij}(s)\rangle_{s}.$$

Hence, the sSCN can be understood as the contribution of each subject to the group-level correlation. The above deconstruction of the sSCN was inspired by a recently introduced edge-centric approach to functional connectivity.^23,24^ In doing so, the morphological information was structured as a 3D matrix 68 [regions of interest (ROIs)] × 68 (ROIs) × 59 (subjects), representing all individual sSCN(s). This derivation of the sSCN allowed us to investigate the relationship of the cortical network with the functional organization derived from the ATM, which had the same 3D structure (see Avalanche estimation) both at the group and individual levels.

### Resting-state EEG recording

The hdEEG recordings were obtained using a 128-channel Micromed System referenced to the vertex. Data were sampled at 1024 Hz and the impedance was kept below 5 kΩ for each sensor. For each participant, we recorded 10 min of closed-eyes resting state while comfortably sitting on a chair in a silent room.

### EEG preprocessing

Signal preprocessing was performed via EEGLAB 14.1.2b.^29^ The continuous EEG signal was first downsampled at 250 Hz and then bandpass-filtered (0.1–45 Hz) using a Hamming windowed sinc finite impulse response filter (filter order = 8250). The signal was visually inspected to identify interictal epileptiform discharges (IEDs) by G.M.D., A.D. and P.B. and then segmented into 1 s long epochs. Epochs containing IEDs activity were removed. Epoched data underwent an automated bad-channel and artefact detection algorithm using the TBT plugin implemented in EEGLAB. This algorithm identified the channels that exceeded a differential average amplitude of 250 μV and marked those channels for rejection. Channels that were marked as bad in more than 30% of all epochs were excluded. Additionally, epochs having more than 10 bad channels were excluded. We automatically detected possible flat channels with the trimOutlier EEGLAB plugin within the lower bound of 1 μV. We rejected an average of 17.34 ± 11.22 (SD) epochs due to spikes and 5.54 ± 3.82 (SD) due to artefacts. The preprocessing analysis pipeline has been applied by our group in previous studies investigating both task-related and resting-state EEG activities.^8,30^ Data cleaning was performed with independent component analysis,^31^ using the Infomax algorithm^32^ implemented in EEGLAB. The resulting 40 independent components were visually inspected and those related to eye blinks, eye movements, muscle and cardiac artefacts were discarded. The remaining components were then projected back to the electrode space. Finally, bad channels were reconstructed with the spherical spline interpolation method.^33^ The data were then re-referenced to the average of all electrodes. At the end of the data preprocessing, each subject had at least 8 min of artefact-free signal.

### Cortical source modelling

The resulting individual surfaces from CAT12 were then imported in Brainstorm,^34^ where three individual surfaces adapted for boundary element models (BEM) were reconstructed (inner skull, outer skull and head) and the cortical mesh was downsampled at 15 002 vertices. The co-registration of the EEG electrodes was performed using Brainstorm, by projecting the EEG sensor positions on the head surface with respect to the fiducial points of the individual MRI. We applied manual correction of the EEG cap on the individual anatomy whenever needed, prior to projecting the electrodes on the individual head surface via Brainstorm. We then derived an EEG forward model using three-shell BEM model (conductivity: 0.33, 0.165, 0.33 S/m; ratio: 1/20)^35^ estimated using OpenMEEG method implemented in Brainstorm.^36^ Finally, we used the weighted minimum norm imaging^37^ as the inverse model, with the Brainstorm’s default parameters setting.

### Avalanche estimation

Similarly to our previous work using neuronal avalanches,^14^ we extracted the activity of a total of 68 ROIs from the Desikan–Killiany atlas.^28^ The ROI time series was obtained by averaging the activity across the vertices composing each ROI. To study the dynamics of brain activity, we estimated ‘neuronal avalanches’ from the source-reconstructed ROI time series. Firstly, the time series of each ROI was discretized, by calculating the *z*-score over time and then setting positive and negative excursions beyond a threshold as 1 and the rest of the signal as 0. A neuronal avalanche begins when, in a sequence of contiguous time bins, at least one ROI is active (i.e. above threshold) and ends when all ROIs are inactive.^11,38,39^ The total number of active ROIs in an avalanche corresponds to its size. These analyses require the time series to be binarized. This is done to ensure that one is capturing critical dynamics, if present. To estimate the suitable time bin length, for each subject, each neuronal avalanches and each time bin duration, the branching parameter **σ** was estimated.^40^ In fact, systems operating at criticality typically display a branching ratio ∼1. The branching ratio is calculated as the geometrically averaged (over all the time bins) ratio of the number of events (activations) between the subsequent time bin (descendants) and that in the current time bin (ancestors) and then averaging it over all the avalanches.^41^ More specifically:


(4) $$\sigma_{i}=\;\prod_{j=1}^{N_{\mathrm{bin}}-1}\;{\left(\frac{n_{\mathrm{events}}\;(j+1)}{n_{\mathrm{events}}\;(j)}\right)}^{\frac{1}{N_{\mathrm{bin}}-1}},$$

(5) $$\sigma =\;\prod_{i=1}^{N_{\mathrm{aval}}}\;(\sigma_{i}{)}^{\frac{1}{N_{\mathrm{aval}}}}\;,$$

where **σ***_i_* is the branching parameter of the *i*-th avalanche in the data set, *N*_bin_ is the total amount of bins in the *i*-th avalanche, *n*_events_ (*j*) is the total number of events active in the *j*-th bin and *N*_aval_ is the total number of avalanche in the data set. In our analyses, the branching ratio was 1 for bin = 2 (corresponding to bins of 8 ms).

### Avalanche transition matrices

An avalanche-specific transition matrix (ATM) was calculated where element (*i*, *j*) represented the probability that region *j* was active at time *t*  *+*  ***σ***, given that region *i* was active at time ***t***, where ***σ*** ∼ 8 ms. The ATMs were averaged element-wise across all avalanches per each participant and finally symmetrized to obtain individualized ATM.

### Statistical analysis

**Figure 1:**
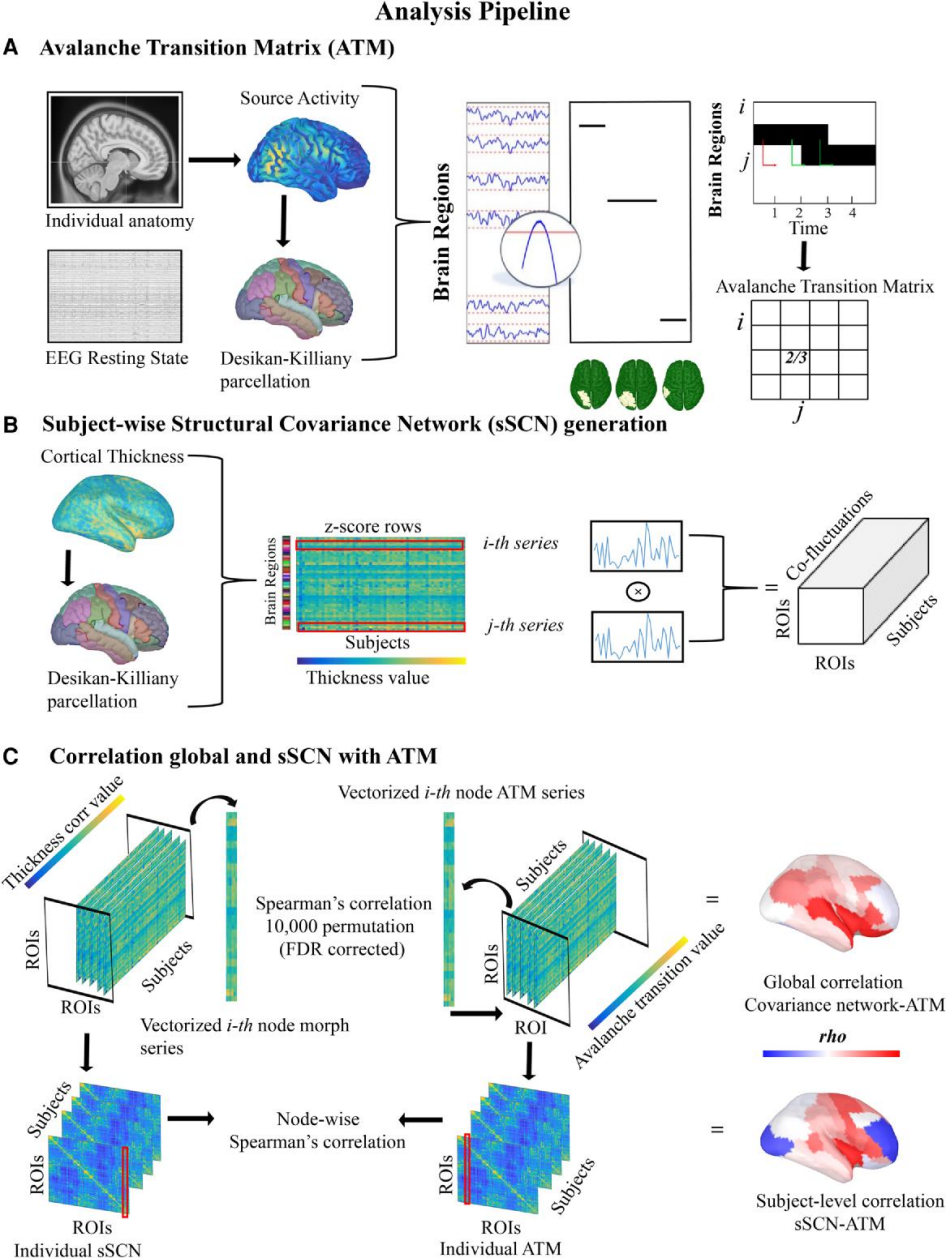
**Analysis pipeline.** The present figure shows the analytical steps in the analysis process. (**A**) Pipeline starting from the structural (MRI) and functional (hdEEG), necessary to obtain the ATM across ROI. (**B**) Computation of the sSCN, from the mean thickness value of the ROI. (**C**) Analytical step in the structure (sSCN)–function (ATM) network correlation both at the group and subject-wise levels.

At first, we correlated the mean thickness value of the ROIs with their mean transitivity (averaged transitivity value of the ROI from ATM) across subjects. Then, we computed the correlation between the network organization of the brain morphology (i.e. sSCN) and the intrinsic functional organization (i.e. ATM). The sSCN matrix was structured similarly to the 3D ATM matrix, i.e. 68 (ROIs) × 68 (ROIs) × 60 (subjects). We then carried out correlations at the global and subject-wise level. Specifically, we extracted the thickness and avalanche transition value from the 3D matrix for each *i*-th node with the rest of the brain across subjects, yielding a 68 (ROIs) × 60 (subjects) matrix. We vectorized these matrices, and we applied permutation-based (10,000 permutation) Spearman’s correlation. The result represents the global correlation of the thickness covariance value and the transition probability of a specific cortical (*i*-th node) area across subjects (see Fig. 1C). Furthermore, it is possible to extract ROIs × ROIs slices from the 3D thickness covariance matrix, which represent the sSCN. We then used Spearman’s correlation between the subject-level sSCN and ATM, obtaining a value between the individual covariance of a specific node and its transition probability (see Fig. 1C). False discovery rate (FDR) was used as a method to correct multiple comparisons^42^ Specifically, we corrected over the node dimension in the group-level analysis and in node × subject dimensions in the subject-wise correlations. We report the corrected *P*-values. Successively, for each region, we provided the percentage of times for a region showing a significant sSCN–ATM relationship across subjects. Finally, we correlated, using Spearman’s correlation, the number of significant regions for each subject with the age of onset, the disease duration and the number of ASMs. Additional analysis was performed to check for potential confounding effects of the focal cortical dysplasia (FCD) and the relationship of structure–function coupling with neuropsychological scores (see Supplementary Table 3).

## Results

### Group-level analysis

**Figure 2:**
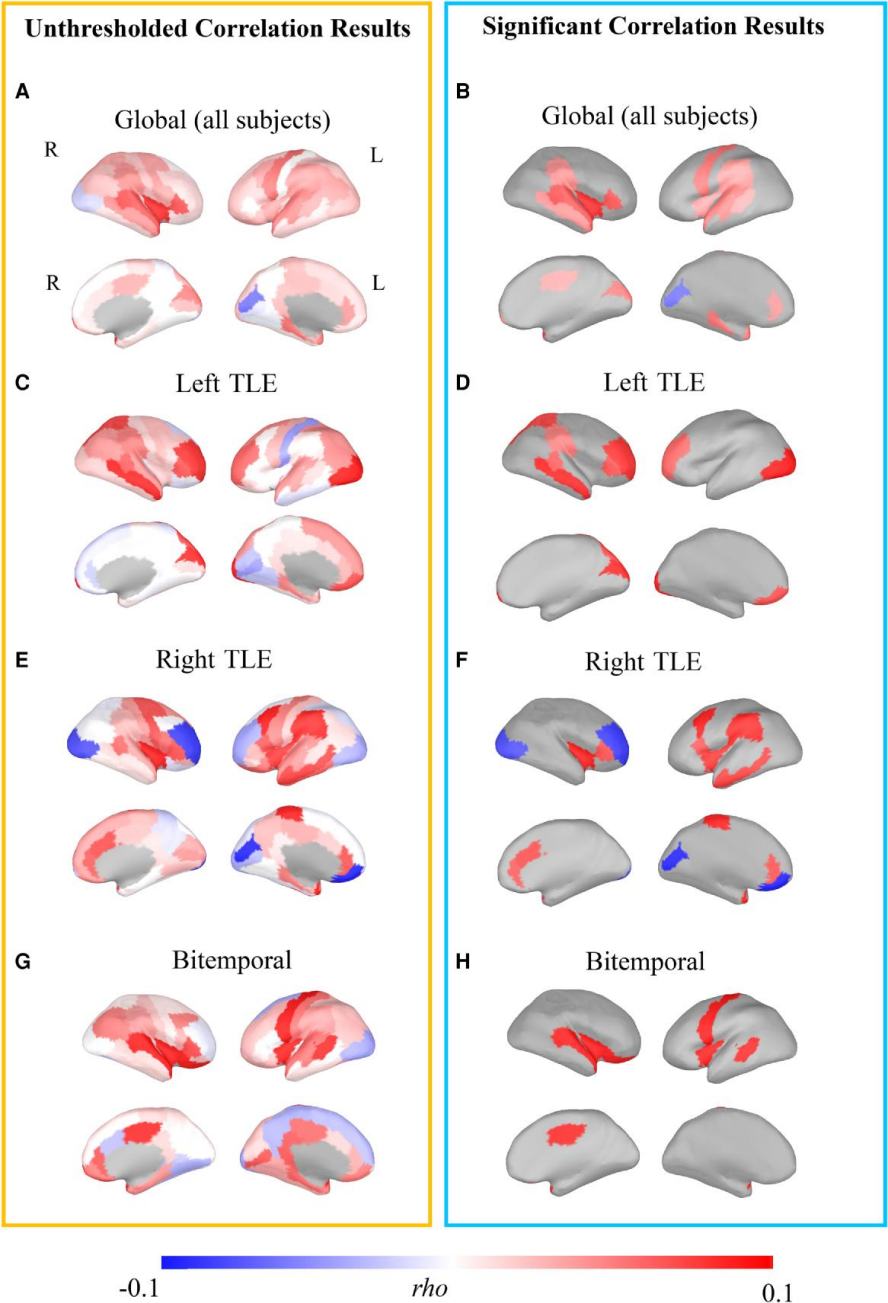
**Group-level structure–function network correlation.** Left panel: untresholded correlational results at the group level between the covariance network and the ATM. Right panel: thresholded (*P*-value < 0.01) results after FDR correction. Specifically, **B** shows the results taking into account the full sample of 59 subjects. Additionally, **D**, **F** and **H** show the group-level results for patients with left TLE, right TLE and bilateral TLE (bitemporal).

For the sake of simplicity, in this section, we report the maximum structure–function correlation Sperman’s *rho* for each group. The *rho* and the relative *P*-value for each significant region and each group are provided in the supplementary materials. When considering the correlation between the average cortical thickness of a ROIs and its average transition probability, no significant results were found (*P*-value > 0.05). Group-wise significant correlation between structure and function was detected when considering the network level both at the structural (thickness co-fluctuation) and functional levels (ATMs). Figure 2 shows the unthresholded (panel A) and the corresponding thresholded (panel B) surface maps (*P*-value < 0.01; FDR-corrected; max *rho* = 0.092; for all the regions and their value, see Supplementary Table 4). One can note that the statistical significance displayed a characteristic topography of the regions where structure and function correlated. In particular, structure–function correlation clustered in the temporal regions, including the superior and middle temporal poles, the insula, the anterior middle cingulate and the parahippocampal cortices (Desikan–Killiany atlas based) (see Fig. 2B). The *rho* value was positive, indicating that larger thickness co-fluctuations in these regions correspond to an increased probability of neuronal avalanche spreading through them. We repeated the analysis for the left TLE, right TLE and also for bitemporal patients separately. On the one hand, our findings highlight a significant contribution of the temporal areas contralateral to the EZ. In the left TLE, we observed a significant correlation (*P*-value < 0.01; FDR-corrected; max *rho* = 0.107; for all the regions and their value, see Supplementary Table 5) between the thickness co-fluctuation and the ATM, mainly involving the right temporal areas, i.e. the banks of the superior temporal sulcus and the middle temporal lobe, as well as the parietal (supramarginal and superior parietal) and the bilateral frontal areas (rostral middle frontal and pars triangularis) (see Fig. 2C and D). On the other hand, right TLE patients are characterized by the involvement of the left middle temporal area, as well as the bilateral insular areas in conjunction with caudal and middle frontal regions (*P*-value < 0.01; FDR-corrected; max *rho* = 0.145; see Fig. 2E and F; for all the regions and their value, see Supplementary Table 6). Finally, the bitemporal group shows a correlation of the bilateral banks of the superior temporal sulcus and the insula, as well as of the superior temporal together with the left precentral and right anterior cingulate (*P*-value < 0.01; FDR-corrected; max *rho* = 0.151; see Fig. 2G and H; for all the regions and their value, see Supplementary Table 7).

### Subject-level results

**Figure 3:**
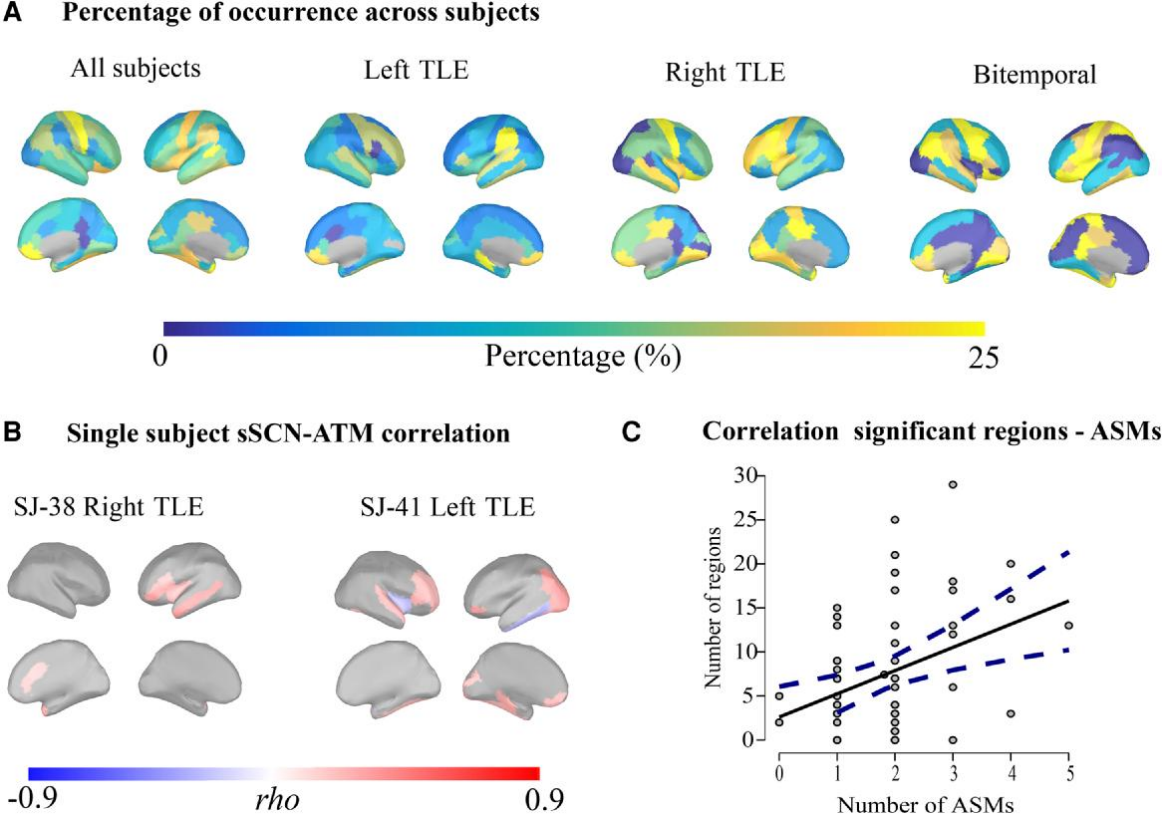
**Additional results.** (**A**) As a summary of the individual-level statistic, the percentage of time across subjects in which a region shows a significant structure–function relationship separately for the four populations, namely all subjects, left TLE, right TLE and bilateral temporal lobe epilepsy (bitemporal). (**B**) sSCN–ATM correlation of two subjects randomly selected (SJ-38 and SJ-41). (**C**) Correlation between the number of regions with a significant sSCN–ATM correlation and the number of ASMs. The lines around the regression line represent the confidence intervals of 95%.

Subject-level results are provided as examples for two randomly selected cases, whereas the maps of all the participants are available online at the following link of the Open Science Framework (https://osf.io/9zj3v/?view_only=94a3287ed35e4040aa791076cc032190). One of the patients reported in the manuscript (SJ-38) is a right TLE patient showing a well-defined sSCN–ATM relationship with involvement of the temporal areas contralateral to the epileptogenic site (max *rho* = 0.448; *P*-value < 0.01; FDR-corrected; see Fig. 3B; for all the regions and their value, see Supplementary Table 8). The second subject that we report (SJ-41) showed a distributed structure–function relationship in bilateral temporal areas, including the parahippocampal cortex, superior and inferior temporal as well as frontal areas (max *rho* = 0.413; *P*-value < 0.01; FDR-corrected; see Fig. 3B; for all the regions and their value, see Supplementary Table 9). As a summary of the subject-wise analysis, we provide the percentage of times across subjects in which a region displays a significant structure–function relationship by region. The temporal and fronto-central areas, as well as the insular and precentral gyrus, the temporal parietal junction and the cingulate cortex (see Fig. 3A), are the most consistent areas with a larger probability of aperiodic bursts propagation in relation to the morphological configuration.

### Relationship with clinical variables

A significant relationship between the number of regions with a significant structure–function link and the number of ASMs was found (*rho*  *=* 0.28, *P*-value *=* 0.030) (see Fig. 3C). In contrast, no significant correlations (*P*-value > 0.05) were found with the age of onset or with epilepsy duration.

## Discussion

In the present work, we investigated the relationship between cortical morphology and brain activity in TLE. We focused on cortical thickness as a marker of brain morphology, as this is altered in TLE.^3,6,43,44^ As for brain activity, we focused on the spontaneous spatio-temporal dynamics from hdEEG data. Specifically, we computed the ATMs that is a mathematical tool accommodating the non-linearities of the large-scale brain dynamics and the corresponding multimodal dynamics that they generate.^11,14,20,21^ Importantly, ATMs were computed over a free signal free of IEDs. Converging evidence showed increased connectivity,^8,9^ as well as structure–function coupling,^45^ during IEDs. The work focused on the investigation of the intrinsic organization of the structure–function coupling, whose alteration may indeed drive to epileptiform phenomena. In a non-free IED signal, if a structure–function correlation was observed, it would have been most likely heavily influenced by IED. In contrast, observing an sSCN–ATM correlation in an IED-free signal allows us to observe the basal configuration of the system. We confirmed our hypothesis that the avalanche spread more across regions that are similar in their structural features, namely the cortical thickness. Larger covariation of thickness across the cortex facilitates/enhances avalanche propagation. These results expand previous knowledge that activity synchronization and propagation depends upon cortical thickness in healthy individuals^46^ by adding the information coming from the non-linearities of the large-scale brain dynamics. It is interesting to note that the relationship between cortical thickness and avalanche spreading involves the limbic system and the regions where temporal lobe seizures are known to typically propagate,^47-49^ such as the bilateral temporal areas, the posterior temporal regions, the insula, the parahippocampal and the cingulate cortex (see Fig. 2B). Interestingly, these are also regions whose cortical thickness has been found to be altered in previous studies, possibly as an effect of the recurrent spread of seizure activity.^4,50,51^ Importantly, the structure–function relationship is not observed when the thickness of a region is directly related to the mean avalanche transitivity but only when the network level is taken into account. These findings imply that the morphology of a single region is not sufficient to explain the spreading of the aperiodic activity by itself. In fact, neuronal avalanches are a product of large-scale brain activity, meaning that the transitivity in one region is highly dependent on the network activity. For this reason, a whole-brain structural configuration perspective can capture the relationship between neuronal large-scale dynamics and brain structure. This is in line with the recent findings that cortical activity can be better understood as resulting from excitations of fundamental, resonant modes of the brain’s structure.^52^ In fact, the sSCN considers the covariance of one region with the others across subjects or intra-individually, partly capturing the geometric configuration of the whole cortex. Our findings suggest that the relationship between structure and activity is significant in both temporal lobes in patients with TLE. To investigate the structure–function relationships in different TLE subgroups, we then performed additional analysis by dividing our clinical population into left, right and bilateral TLE. This comes with the price of a smaller sample size for each group. This analysis revealed that patients with bilateral TLE show a significant bilateral temporal thickness–avalanches relationship (see Fig. 2H). Surprisingly, unilateral TLE was associated with a stronger structure–function link in the contralateral temporal lobe, together with contralateral temporo-parietal junction and bilateral prefrontal areas (see Fig. 2D and F). The thinning of the contralateral temporal areas has been described in unilateral temporal lobe epilepsy with a greater involvement of the right TLE^53,54^ but to a lesser degree as compared with the ipsilateral cortex. One possibility is therefore that the contralateral temporal lobe is less affected by cortical thinning, resulting in an enhancement of the avalanche spread. One alternative explanation is that the contralateral compensatory plastic changes occur in TLE and explain the increased connectivity in the contralateral regions.^55^ Overall, our results align well with the interpretation of epilepsy as a network disorder. While previous studies have highlighted either impairment of structure or functional data, here, we demonstrate how these two properties closely interact, providing a comprehensive view leveraging on multimodal data. We further tried to account for variability in our sample by pushing forward an attempt to observe the structure–function relationship at the subject-wise level, to better take in account the contribution of each patient (see Fig. 3B). Subject-level analysis confirmed and strengthened group-level results. Importantly, we observed that specific cortical regions were consistently recruited more often, across subjects, by ongoing avalanches, particularly in the brain regions that are known to be structurally altered in TLE (Fig. 3A). Finally, with regard to the possible relation between anatomo-functional architecture and clinical variables (age of epilepsy onset, epilepsy duration and number of ASMs), we only found an association between the number of regions with significant structure–function correlation and ASMs, with the former which was increased in individuals with higher ASM load. The ASM load may reflect epilepsy severity, which in turn has been associated with a more diffuse spreading of epileptic activity within the brain.^56^ Moreover, our previous findings highlighted that patients with TLE are characterized by an hyper-integration of the functional networks.^30^ In light of this, we may speculate that a more severe clinical picture, likely requiring a larger number of ASMs, is linked to a widespread dysregulation of both the functional and structural networks, resulting in a less segregated and localized structure–function links. To sum up, in the present work, we merge structural and functional imaging in TLE patients and show a relationship between the alterations of the aperiodic dynamics and cortical organization. We provided a methodological insight with a new way to compute an sSCN, together with an innovative approach investigating the aperiodic brain dynamics, namely the neuronal avalanches. Neuronal avalanches are typically used within the framework of criticality, which describes the collective behaviour of neural assemblies at different spatial scales.^57,58^ Neural networks operating near criticality are linked to optimal information processing,^59,60^ making this measure a potential biomarker both for altered neurophysiological^61^ and neuropsychological^62^ functioning. Alterations of the critical dynamics are related to the initiation and the termination processes of seizures, providing useful information of the mechanisms involved.^17,63^ In the light of this, the ATMs represent a useful and novel approach mining into the large-scale network dynamics, which are known to be altered and strictly linked to the pathophysiological processes in epilepsy. However, although alterations of ATMs in TLE was evidenced in our previous study on a subpart of the present sample, the lack of control group does not inform us whether the structure–function relationship we have observed is specific to epileptic patients or, rather, it also exists, at least to some extent, in healthy subjects. Overall, our approach finds its rationale in the idea that the altered activities in epilepsy might be the result of local structural alterations and the way they affect the resonances that are generated at the whole-brain level. As such, the integration of structural and whole-brain functional data is indispensable. Our findings could represent a first evidence demonstrating the importance of the inclusion of the morphological heterogeneity across regions in order to increase the accuracy of the models of brain dynamics in epilepsy,^25,64,65^ as suggested by Suarez and colleagues.^12^ In fact, the inclusion of the morphological configuration to predict the behaviour of the brain networks is useful for the diagnosis of epilepsy, as well as to enhance modelling for the optimization of drug delivery or neuromodulatory approaches.^66^

## Conclusions

In the present work, we deployed a novel methodological approach to test the hypothesis that large-scale dynamics is influenced by structural features of the cortex. We observed a stable cluster of correlation in the bilateral temporal and limbic areas across subjects, highlighting group-specific features for left, right and bilateral TLE patients. We developed strategies to bring the investigation to the individual level, confirming group-wise findings and expanding them to the single-subject level. We confirmed that TLE is characterized by structural cortical alterations that are intimately related to the alteration of the fast whole-brain functional dynamics. Finally, we showed that the structure–function link has a tight relationship with clinical features such as disease severity. In this study, we leveraged on a well-defined model of neurological disease, namely the TLE. Nevertheless, the present methodology may have potential implications also in other conditions characterized by structural alterations across the brain (e.g. stroke and multiple sclerosis).

## Supplementary material

Supplementary material is available at *Brain Communications* online.

## Supplementary Material

fcad348_Supplementary_Data

## Data Availability

The data that support the findings of this study are available on request from the corresponding author. The raw data are not publicly available due to privacy or ethical restrictions.
