# Supplementary material for: Altered spread of waves of activities at large scale is influenced by cortical thickness organization in temporal lobe epilepsy: a magnetic resonance imaging–high-density electroencephalography study
Source: Brain Commun. 2023 Dec 14;6(1):fcad348. doi: 10.1093/braincomms/fcad348 (PMC10754317; doi:10.1093/braincomms/fcad348)
Supplement: fcad348_Supplementary_Data [file fcad348_supplementary_data.pdf]

SUPPLEMENTARY MATERIALS

MRI acquisition

**Sequence**	**Field strength**	**Slice thickness**	**TR**	**TE**	**Flip angle**
MPR BRAVO 1mm	3T Signa Pioneer	1	8.9ms	3.2ms	12
PSPGR BRAVO 1mm	3T Signa hdxt	1	7.7ms	2.9ms	12
MPRage 0.9mm	3T Siemens	0.9		2.4ms	9
SAG 3D T1	3T Philiphs	1	7.8ms	3.5ms	8
FL3D	1.5T Siemens	1	9.5ms	4.7ms	20
sT1w 3D MTC RIC	1.5T Philiphs	1	7 ms	3.1ms	8

Supplementary Table 1. Image acquisition details

Neuropsychological Testing

Patients with TLE in the center generally undergo a neuropsychological evaluation focusing on global intelligence, memory and attention/executive functions. Specifically, the total IQ (TIQ) of the WAIS-IV scale (Orsini & Pezzuti, 2013) is used as a global intelligence measure. Memory is evaluated via digit span; spatial short/long term memory is investigated with the delay recall of the Rey–Osterrieth Complex Figure Test (ROCFT) (Caffarra et al., 2000) while verbal short/long term memory is evaluated via Rey Auditory Verbal Learning Test. Attention and executive functions are evaluated with the Trail Making Test (TMT). In this retrospective cohort there is a large inhomogeneity across patients of the present score of the neuropsychological test. Only 24 subjects have a full assessment battery. A description of the mean value and standard deviation for each test score of this subsample is provided in Supplementary Table2.

**Neuropsychological test**	**Mean ± standard deviation**
Total IQ WAIS-IV	91.041 ± 18.767
Digit span	5.625 ± .769
ROCTF - copy	33.095 ± 3.419
ROCTF - reproduction	17.666 ± 6.332
RAVLT - immediate	37.333 ± 9.291
RAVLT - delay	8.00 ± 6.082
TMT-A	34.80 ± 17.374
TMT-B	121.666 ± 86.959

Supplementary Table 2. Mean score and standard deviation of the neuropsychological tests scores.

Additional correlation analyses

In order to check for the potential confounding effect of the presence of focal cortical dysplasia over the correlational results, we performed a two-tailed unpaired t-test on the number of regions displaying structure-function correlations in patients with FCD vs. the rest of the sample. We did not evidence significant results $t\ (57) = -1.690$; $p = .096$, and thus supports a negligible effect of the FCD on the thickness computation in our sample. Additionally, we performed Spearman's correlation between the number of regions with significant structure-function correlation and the neuropsychological tests score. No significant results were found. We provide in the following table (Supplementary Tab.2) the *rho* and the uncorrected *p* values.

**Neuropsychological test**	**Number of regions**	
	rho	***p-value***
ROCFT copy	.324	.122
ROCFT reproduction	.057	.790
QIT	- .017	.935
TMTA	-.003	.989

TMTB	-.187	.394
RAVLT immediate	.396	.055
RAVLT delay	.264	.213
Digit span	.231	.278

Supplementary Table 3 Correlation results between the number of regions with significant structure-function relationship and the neuropsychological tests scores.

**Regions**	***rho***	***p*-val**
bankssts L	0.0427	0.0072
bankssts R	0.0781	1.00e-6
cuneus L	-0.0642	0.0004
cuneus R	0.0630	1.00e-6
frontalpole R	0.0546	1.00e-6
insula L	0.0438	0.0048
insula R	0.0922	1.00e-6
middletemporal R	0.0486	0.0020
parahippocampal L	0.0607	1.00e-6
parstriangularis R	0.0768	1.00e-6
posteriorcingulate R	0.0447	0.0060
precentral L	0.0597	1.00e-6
rostralanteriorcingulate L	0.0498	0.0016
superiortemporal L	0.0553	0.0004
superiortemporal R	0.0726	1.00e-6
supramarginal L	0.0450	0.0044
supramarginal R	0.0485	0.0036
temporalpole L	0.0555	1.00e-6

Supplementary Table 4. Significant regions with the corresponding Spearman's *rho* and *p*-value for the whole-group analysis.

Regions	*rho*	*p*-val
bankssts R	0.0725	0.0008
cuneus R	0.0994	1.00e-6
frontalpole L	0.0771	0.0004
frontalpole R	0.0957	0.0003
lateraloccipital L	0.1071	1.00e-6
medialorbitofrontal L	0.0806	1.00e-6
middletemporal R	0.0968	1.00e-6
parstriangularis R	0.0763	0.0016
rostralmiddlefrontal L	0.0669	0.0028
rostralmiddlefrontal R	0.0887	0.0008
superiorparietal R	0.0818	1.00e-6
supramarginal R	0.0557	0.0096

Supplementary Table 5. Significant regions with the corresponding Spearman's *rho* and *p*-value for the left-TLE group analysis.

Regions	*rho*	*p*-val
caudalanteriorcingulate R	0.0861	0.0028
caudalmiddlefrontal L	0.0983	1.00e-6
cuneus L	-0.1526	1.00e-6
insula L	0.0902	0.0012
insula R	0.1454	1.00e-6
lateraloccipital R	-0.0773	0.0080
medialorbitofrontal L	-0.1205	1.00e-6
middletemporal L	0.0847	0.0024
paracentral L	0.1273	1.00e-6
parsopercularis L	0.0781	0.0076
parstriangularis R	0.0790	0.0048
rostralanteriorcingulate L	0.0806	0.0032
rostralanteriorcingulate R	0.0798	0.0048
rostralmiddlefrontal R	-0.0856	0.0020
supramarginal L	0.0948	0.0004
temporalpole L	0.1033	0.0004

Supplementary Table 6. Significant regions with the corresponding Spearman's *rho* and *p*-value for the right-TLE group analysis.

Regions	*rho*	*p*-val
bankssts L	0.0987	0.0012
bankssts R	0.1009	0.0020
insula L	0.1055	0.0012
insula R	0.1359	1.00e-6
lateralorbitofrontal R	0.1087	0.0016
posteriorcingulate R	0.1268	1.00e-6
precentral L	0.1160	0.0012
superiortemporal R	0.1518	1.00e-6

Supplementary Table 7. Significant regions with the corresponding Spearman's *rho* and *p*-value for the bilateral TLE group analysis.

Regions	*rho*	*p*-val
insula L	0.3282	0.0066
middletemporal L	0.4483	0.0001
parsopercularis L	0.3142	0.0095
parsorbitalis L	0.3862	0.0012
parstriangularis L	0.3724	0.0019
rostralanteriorcingulate R	0.3230	0.0076
temporalpole R	0.3847	0.0013

Supplementary Table 8. Significant regions with the corresponding Spearman's *rho* and *p*-value for the individual level analysis example subject-38.

Regions	*rho*	*p*-val
fusiform L	0.3181	0.0086
inferiorparietal L	0.4133	0.0005
lateraloccipital L	0.3972	0.0008
medialorbitofrontal L	0.3304	0.0063
parahippocampal L	0.3800	0.0015
parsorbitalis L	0.4031	0.0007
parstriangularis R	0.3315	0.0061
superiortemporal R	0.3262	0.0070

Supplementary Table 9. Significant regions with the corresponding Spearman's *rho* and *p*-value for the individual level analysis example subject-41.
